# Sport Cyberpsychology in Action During the COVID-19 Pandemic (Opportunities, Challenges, and Future Possibilities): A Narrative Review

**DOI:** 10.3389/fpsyg.2021.621283

**Published:** 2021-03-03

**Authors:** Olivia A. Hurley

**Affiliations:** Department of Technology and Psychology, Institute of Art, Design, and Technology, Dublin, Ireland

**Keywords:** sport cyberpsychology, COVID-19, technology, pandemic, social media, video analysis, GPS, online consulting

## Abstract

Interest in sport cyberpsychology has become more popular over the last decade, primarily due to the increased use of technology and the online world, including social media, within sport settings (Hurley, 2018). In 2020, this became even more apparent for many athletes, their support teams and their sport organisations, when their professional and social worlds became cyber-dominated due to the COVID-19 pandemic. Many challenges were encountered by: (i) the athletes, in their efforts to remain active and well during this time when all competitions were cancelled and (ii) the healthcare professionals, working with these athletes, in their efforts to continue to support the athletes, when severe travel restrictions and social distancing were in place for considerable periods of time. The purpose of this paper, using a narrative review process, is to present and scrutinise an array of Internet interventions, remote psychological supports and applications (apps) that the athletes and their support personnel used to help them meet their physical, social, and emotional needs during the pandemic. The beneficial and restrictive features of these online options will be presented. Two main themes will be considered in order to focus this discussion, namely, (i) the potential physical and mental opportunities and challenges using the online world extensively during this time presented for the individuals working in sport and (ii) suggestions for how such online interventions used by the athletes, their coaches and sport science personnel during the pandemic may be maintained in some positive ways into the future, to help the athletes prepare for their upcoming competitions, their training and their future careers when they retire from elite sport.

## Introduction

Competing at major sport events, such as an Olympic Games, are often the culmination of years of preparation and competition for many athletes. However, in 2020, the COVID-19 pandemic halted competitive sport across the globe, much to the distress and disappointment of these athletes, their support personnel, sport organisations and the sponsors of these events (Hytner and Butler, 2020; McCurry and Ingle, 2020; O’Connor, 2020; RTE, 2020). Their worlds, like so many other groups, also became dominated by online interactions. This has resulted in some major challenges for many of these athletes, such as: (i) how to identify themselves, when they were not able to compete and earn a living from their sport, and (ii) how to remain active, fit and holistically well when their training venues were closed and they were forced to spend long periods of time in isolation, like so many other populations (Schinke et al., 2020).

The potential negative health risks, excluding the threat of contracting covid-19 itself that the imposed restrictions and necessary isolation periods presented for many of these individuals have been, in some ways, similar to the general population (Schinke et al., 2020). For example, increased sedentary behaviours [see Chtourou et al. (2020) for a comprehensive review of the importance of physically activity during the pandemic], poorer dietary choices and unhealthy levels of alcohol consumption are coping strategies frequently linked with lower levels of physical and psychological well-being (Ingram et al., 2020; López-Bueno et al., 2020) and like many groups during the pandemic, athletes may also have employed some of these coping strategies when prevented from training daily with their peers in their typically disciplined environments. Also, within the general population, for which athletes make up a proportion, increased levels of domestic abuse, violence and mental health distress, including suicide rates, have been reported throughout the pandemic (Schinke et al., 2020). However, athletes may have experienced specific challenges unique to their cohort too, such as various forms of mental health distress (Davis et al., 2020; FIFPRO, 2020; Hull et al., 2020) and the pressure of public expectations (Foskett and Longstaff, 2018) to be national and international ‘leaders’ in promoting healthy coping strategies throughout the pandemic. They may have felt obliged to take part in various sport challenges, as well as to communicate public health messages to their, typically large, fan-base, using their online social media platforms, for example (Sharpe et al., 2020).

In contrast, however, some athletes may have opportunistically used the ‘lock-down’ time during the pandemic in a positive way, to ‘upskill’ perhaps in preparation for their lives after sport (MacIntyre et al., 2020; Schinke et al., 2020). However, engaging in these activities for some athletes may have also triggered an enforced acknowledgment of the precarious nature of a sport career (Schinke et al., 2020). That said, in studying the impact of the pandemic, both personally and professionally on Spanish Olympic and Paralympic athletes, Clemente-Suárez et al. (2020) reported that some athletes appear to have coped well when restrictions were placed upon them. Clemente-Suárez et al. (2020) proposed that this was, perhaps, due to these athletes’ additional cognitive resources and abilities that they could call upon from their specific, sport-developed, and coping skills. Further, they reported that the athletes who participated in their study appeared to remain within non-pathological parameters for anxiety during the pandemic. They also noted that the educational status of the athletes in their study appeared to play a role in their ability to develop psychological flexibility during the pandemic, with third level (college/university) educated athletes appearing to display this more than their high-school educated counterparts.

Psychological flexibility refers to the ability to be able to positively adapt to changing situations as they arise (Clemente-Suárez et al., 2020). Applying this ability has been shown to psychologically benefit a number of populations throughout the pandemic (Landi et al., 2020). A large part of the challenge facing many athletes has also been applying such an ability throughout 2020. Their additional skills, such as their typical levels of conscientiousness and effective scheduling of their daily activities to help them maintain their athletic performances prior to 2020, could also have helped them to manage their disrupted lives when the pandemic began. To explain, while as human beings athletes are no less potentially prone to distractions and disruptions in their daily routines as other members of the general population are, many of them do train themselves to respond in positive ways to such situations and to be solution-focused when they occur. Thus, many athletes may have aimed to maintain some form of that ‘normality’ throughout 2020 by using their routine-management skills (Pierce et al., 2018; Kendellen and Camiré, 2019). For many of these athletes’ support providers, helping them to achieve this sense of some control and ‘normality’ over their lives during the pandemic was also a likely priority (Earley, 2020a,b; MacIntyre et al., 2020; Whales et al., 2020). From such a support personnel perspective, how to advise and help these athletes, while severe travel restrictions and social distancing has remained in place throughout 2020 has been challenging. Technology use has been at the core of the solution-focused strategies used by many athletes and their support providers, as it has been for other service providers (Seshadri et al., 2020) dealing mainly with the general public, where patient monitoring has been required (for example, medical consultants, general practitioners (GPs) and physiotherapists). Luckily, in recent years, many athletes and their support scientists have worked together to produce advanced technologies that effectively record training data and allow them to communicate remotely when necessary (Hurley, 2018). Never have such developments been more potentially valuable than during the 2020 coronavirus pandemic. So, with all of the above in mind, questions such as: (i) what tools and techniques, with the help of some of these advanced technologies, have the athletes and their support personnel relied upon to help them to continue to make progress with their training and service provision throughout the pandemic, (ii) what performance and data monitoring, even when in enforced isolation and travel restriction situations, have they been able to apply effectively and (iii) what benefits and limitations do such technology-based performance, health and lifestyle data systems offer to athletes, especially considering their mental health needs during this time of great uncertainty throughout 2020, are worth exploring. Thus, the purpose of this paper is to consider, and provide some answers to the above questions, by completing a narrative review of research in the relevant areas. An array of technology-based-internet interventions, remote psychological supports and applications (apps) that athletes and their support personnel have used during the pandemic to help them to meet their physical, social, and emotional needs will be discussed. The information will be presented under the following nine main headings: (i) A brief overview of the ‘scene,’ including an explanation of the term sport cyberpsychology, (ii) video analysis technology use, (iii) virtual reality technology use (iv) global positioning system (GPS) technology use, (v) wearable applications (apps), that monitor various relevant activity levels, sleep behaviours and calendar/scheduling features, for example, (vi) online sport psychology consulting during the pandemic (benefits and threats), (vii) online delivery of training and coach development during the pandemic, (viii) athletes’ use of social media during the pandemic and, finally, (ix) some conclusions and final thoughts, including future research possibilities moving forward to the end of 2020 and into 2021.

## A Brief Overview of the ‘Scene’ – Introducing the Term Sport Cyberpsychology

Before the various technologies used are outlined, a relevant psychological term, sport cyberpsychology, should first be explained. In simple terms, sport cyberpsychology could be described as the study of cyberpsychology within sport settings, which prompts the question: what is cyberpsychology? In brief, Kirwan (2016a) described the discipline as: (i) the study of how people interact with each other when using technology, (ii) technology development for the benefit of users’ requirements, and (iii) the examination of how technology specifically impacts psychological states and behaviour. Hurley (2018) proposed sport cyberpsychology to be the study of how humans interact with various technologies, including the Internet, mobile devices, virtual reality and gaming systems within sport settings, and how such technologies, including social media use, for example, impact athlete behaviour and well-being. Over the past two years especially, a number of studies have supported the previously, and predominantly anecdotal evidence, that various new technologies used within sport settings are enabling athletes to prepare for, and compete better in, their sport (Figueira et al., 2018; Torres-Luque et al., 2018; Bedir and Erhan, 2020; Lund, 2020). Technologies using virtual reality and GPS tracking, as well as various mobile apps are constantly being designed to give more accurate, objective measures to athletes and their coaches regarding their performances (Earley, 2020a,b), as well as monitoring and providing them with feedback on their recovery status (i.e., their sleep quality, sleep quantity and heart rates; Peake et al., 2018). In the world of sport, where competitive performances are typically based upon very objectively measured, numerical data (rather than performance judgements of others, which notably are still in place in sports such as boxing, gymnastics and diving, for example), athletes and their support personnel have responded enthusiastically to the emergence of these new technologies (Farrell, 2019). The reason why is perhaps because they can provide them with more objective data, that gives them specific information on aspects of their training and recovery that historically could only be determined, to some degree, via subjective and, therefore, somewhat less reliable, self-report measures (Robson, 2016; Smith, 2017). Of course, many of the available technologies (mental well-being apps, for example) can also be used by athletes and their support staff to continue to document and record their psychological states, such as their perceptions of their emotions, on a daily basis. Many teams gather such helpful data from their athletes in order to monitor their well-being and in order for them to decide how best to continue supporting the athletes. Some of the benefits and limitations of these specific types of technologies athletes and their support personnel have been using during 2020 to help them maintain their training standards and general wellbeing, both physically and mentally, will now be considered.

## Video Analysis Technology Use

The use of video technology to record and analyse the movements of athletes as they train and compete in real-time in order for often-specialised sport scientists (typically biomechanics experts) to identify areas for technique improvement, has been in place for a number of years (Hurley, 2018). The science of biomechanics has become an integral part of sport performance, not only for performance benefits but also, importantly, for injury prevention and rehabilitation purposes (McGinnis, 2013). However, video analysis has also been introduced into various sport settings for other beneficial purposes, such as into stadia to help officials make on-field decisions regarding the scoring of goals, tries or points. They also help them to review and adjudicate potential illegal actions by athletes during competitive events and then whether or not to issue various penalties, such as red, yellow or black cards in sports such as soccer, rugby, tennis, basketball and Gaelic football for example (van Biemen et al., 2018; Sors et al., 2019). Video play back has also become part of the normal spectator and fan-experience when watching sport, either from their homes or in person in stadia during breaks in competition or play (Arkenberg et al., 2019).

Throughout the pandemic, athletes have been able to use their smartphone devices’ camera function to video record their own training sessions (Whales et al., 2020). They could then share these recordings after or indeed, ‘live,’ with their coaches and sport science personnel (as well as with their social media followers quite often throughout 2020). Such mobile technology is very useful for the athletes and their support teams because it can be employed anywhere. Its use is not confined to the formal training venue as may have been the case in the past. Some research studies have identified both the strengths and challenges this technology presents in modern day sport (Groom, 2012). Various physical and mental benefits of using video technology during the pandemic for athletes and their coaches could have included: (i) the objective nature of the data recorded – it is real and evident for all to see, (ii) its ability to help keep the athletes accountable and motivated to train (for example, if such video evidence of completing training sessions was requested by coaches and the athletes’ various support staff), (iii) its potential ability to improve athletes’ self-efficacy (self-belief) and mental imagery skills (Buck et al., 2016), as well as (iv) its use in positively aiding their decision-making skills (Pagé et al., 2019) and pre-performance routines (Cotterill, 2018). However, the challenges of using such technology can also include: (i) the pressure it potentially places on athletes who may not feel comfortable recording themselves on camera, (ii) the sense they may have that their training is always being observed and scrutinised rather than them being trusted to complete various sessions without such objective video evidence (Ogden, 2011), and (iii) the feelings of guilt or disappointment they may experience at not ‘being perfect’ in their execution of their skills when they watch-back the video evidence of their performances (Middlemas and Harwood, 2017; Harris, 2018). These are all issues that individuals supporting athletes should be aware of and discuss with them in advance of requiring or encouraging such video recordings of athletes’ training sessions. Such discussions can ensure a best-practise, athlete-centred approach, regarding how to proceed or how to modify training sessions for the duration of the pandemic, to the satisfaction of all concerned. This approach is more likely to help maintain the desirable ‘facilitative’ sport environment, where athletes are challenged and are also well-supported (Sarkar, 2018).

## Virtual Reality Technology Use

Virtual reality and immersive technology advances have enabled athletes to train in simulated environments when they cannot train in their real-life environments due to, for example, adverse weather conditions, injury or the very nature of the sport (Farley et al., 2020). Training of medical, military and aviation personnel using this technology has also been beneficial in recent decades (Langfield, 2019). The use of virtual reality and immersive video technology to enable athletes to specifically practise their reaction time skills, their decision making skills as well as their concentration skills when they are not able to compete, such as when rehabilitating from injury, for example, has been a positive application of this technology for a number of years (Panchuk et al., 2018; Farley et al., 2020). VR technology has also been used effectively to treat clinical conditions including anxiety, paranoia and phobias across various populations (Coyle et al., 2011; Gega et al., 2013; Flood, 2016; Kirwan, 2016b).

During 2020, VR technology could have offered a way for athletes to immerse themselves in some form of their sport environment that mimicked their ‘real’ competitive worlds, including the use of gaming options perhaps (Hurley, 2016; Kirwan, 2016b), when they could not physically experience them due to the suspension of all sport, especially in the earlier months of 2020. They could also have used VR technology for the purposes of computerised cognitive behavioural therapy (cCBT; Twomey et al., 2013), if required, as such computerised therapy can be therapist-lead or self-lead. Both could have been good options to have access to during the pandemic, when in-person CBT was frequently not feasible for psychologically distressed individuals, including those from within sport settings. However, while VR technology can be somewhat beneficial to athletes for the reasons stated above, the feasibility of using such technology during the pandemic, when most athletes would not have been able to access the most sophisticated versions of such technology in their own homes, for example, is a main limitation of such VR training. It is also important to note that even the most advanced VR devices and advances in haptic technology do not exactly mimic the performance setting (Wang et al., 2019). This technology, that attempts to create kinaesthetic communication or ‘3D touch’ by applying forces, vibrations and motions to the user remains limited. For example, its ability to recreate the exact ‘reality’ of the setting and experiences which athletes sense and perceive when they train and compete, physically is restricted. Further research on such features of VR technology would be welcomed across many sport-specific and other performance settings (in surgery, for example, where VR training has been used extensively to training and assess surgeons’ psychomotor skills; Hofstad et al., 2013).

## Global Positioning System (GPS) Technology Use

Global positioning system technology has revolutionised the sport performance industry. This satellite supported system allows the movements of individuals and objects (such as cars) to be detected. GPS technology typically measures the velocity, direction and speed of performers, making it effective technology for team coaches, strength and conditioning, and medical personnel, who design the training programs of the athletes to have access to The Hard Yard 2017). It can also help them to make decisions regarding athlete selections and substitutions, even in real time during matches or competitions (Earley, 2020a). Most elite athletes and teams use some form of this GPS technology to assist them in their training. In 2020 many companies have been actively assisting athletes to train remotely using their devices (Earley, 2020b). These companies have designed fitness wearable performance devices (see below for more details on these) incorporating elements of GPS technology, to enable the athletes and their coaches to accurately track specific aspects of their training. See Dellaserra et al. (2013) and Earley, 2020a,b for a review of some of the uses of various integrated technologies in team sports, including GPS devices). Such technology has been found to be effective in its testing and recording of performance markers of strength, power and flexibility, all of which are important measures to have in regard to rehabilitation from injury and for performance enhancement potentially.

## Wearable Apps – Activity Levels, Sleep Behaviours, Psychological State Monitoring and Calendar/Scheduling Features

Using wearable devices to monitor physical activity levels, psychological states, heart rates, sleep, diet, and recovery has been an area of research interest for a number of years, likely due to the dramatic increase in the number of individuals employing them for such purposes in their training and daily lives (Peake et al., 2018). The use of activity tracking apps to encourage beneficial levels of physical activity among elite and non-elite populations have been more extensively and scientifically studied in recent years also (Peake et al., 2018). This is a welcome development as initially most of the available research examining the strengths and limitations, even dangers perhaps, of such app use was somewhat questionable. This was, perhaps, due to such research often being commissioned by the companies developing and selling the apps. These companies would, understandably, wish for them to be viewed in a mainly positive and beneficial way. However, this can also result in potential conflicts of interest when interpreting the results from such studies (Peake et al., 2018). However, companies and researchers have improved their methods of research in recent years and the general public has also become better informed regarding the different available products and their limitations, as well as their strengths. For example, a positive application of such wearable devices is that they may help people to develop autonomy, self-efficacy, and accountability strategies for engaging in their own physical activities. However, negative outcomes of their use in regard to such constructs in some specific populations have also been reported. For example, Kerner and Goodyear (2017) examined the use of wearable health technologies (Fitbit) by adolescents, in the context of the behaviour change, self-determination, theory (SDT; Deci and Ryan, 1985, 2000, 2008). SDT strives “to explain human motivation and behaviour based on individual differences in motivational orientations, contextual influences on motivation, and interpersonal perceptions” (Hagger and Chratzisarantis, 2008, p. 80). SDT assumes that individuals seek out challenges and are intrinsically motivated, that is, they are growth-oriented, they seek out activities they are interested in and find inherently enjoyable (Standage et al., 2014). SDT proposes that the greatest well-being and performance benefits are reportedly experienced when individuals’ needs for relatedness (feelings of closeness and being connected with others as part of a community), autonomy (feeling in control of personal actions), and competence (feeling capable of being able to complete desired activities using available skills) are met. The young participants in Kerner and Goodyear’s (2017) study reported feelings of pressure to compete with their peers in the activity challenges set for them as part of the study, as well as feelings of guilt if those targets were not achieved. Their need satisfaction and autonomous motivation reduced as the 8-week study progressed while their amotivation increased.

In contrast to Kerner and Goodyear (2017) and Kononova et al. (2019) examined the use of activity tracker use among older adults, in varying stages of their use of the technology (non-use, short-term use, long-term use and abandoned use). They considered the older adults’ behaviours regarding their physical activity engagement, using the tracker-technology, in the context of a different behaviour change model, that of the transtheoretical model of behaviour change (TTM; Prochaska and DiClemente, 1983). This stage-like model regards behaviour change as happening gradually, over time, by progressing through a series of stages or processes labelled as: pre-contemplation, contemplation, preparation, action and maintenance. Additional stages such as termination and relapse have also been considered as part of this model. Kononova et al. (2019) reported that intrinsic motivation and social support were important in determining the longer-term use of such tracking devices by older adults. They remarked that such beneficial technology is underused among these populations, that younger populations are often the target groups for such activity apps, despite the importance of physical activity in helping to prevent diseases more prevalent and associated with aging populations. This is an area for potentially exciting practical implications in the coming years.

During the pandemic, the activity tracking apps referred to above, that monitored individuals’ daily step allocation for example, appeared helpful to encourage people across many cohorts to stay fit and active, even when their usual travel and movements were severely restricted. As alluded to above, the social support communities that such activity apps can create and provide for users can be effective (Petersen et al., 2020). Some of the specific beneficial features of this technology can include the motivational and reminder messages the apps communicate to their users. However, the advice offered by such devices can also be somewhat arbitrary and, at times, meaningless and non-sensical. For example, why is 10,000 daily steps seen as a meaningful measure of health? Despite this metric being a widely reported and used target for health benefits and maintenance, research support for this activity target is surprisingly lacking (Hammond, 2019; Lee et al., 2019). Thus, the notes of caution for such device-use should also be acknowledged. Specifically, for example, within elite sport settings their use could result in some individuals feeling under pressure to engage in excessive levels of activity, if they become ‘too’ competitive with others in their social or competitive community, or even with themselves, if they aim to constantly ‘beat’ their daily personal bests (PBs). Such behaviours may then lead to injuries, burnout and psychological distress. Perhaps weekly activity ‘ranges’ or ‘zoned’ targets such individuals could be encouraged to reach may be a more positive strategy to apply in these contexts. Rest days, where a lower activity load is applied is factored into the activity schedules of athletes. This is important to enable the athletes’ bodies to recover from their high training loads. Recovery emphasis and individual tailoring of activity programs should be a focus of support staffs’ discussions with their athletes regarding activity tracker use, to help prevent the pressure of ‘having’ to be achieve constant PBs being experienced by the athletes. Kerner and Goodyear (2017) recommended that personalised health targets should be encouraged when aiming to support young people, athlete or not, to personally and critically engage in health-based activities. Kononova et al. (2019) also suggested that designers of these technologies apply a multi-factor approach (at an individual-level, interpersonal-level and community-level) to ensure varying populations maintain the recommended and generally regarded appropriate physical activity targets with the assistance of the available technology based activity trackers.

Similar to physical activity monitoring, sleep monitoring has become a feature of interest for many athletes to track also. While understandable as a feature of curiosity in their daily lives, especially considering the comments above regarding the importance of adequate rest and recovery for such individuals, it can also be paradoxically unhelpful, for some people. If the recorded data on their app indicates they are not appearing to spend the recommended 7 h to 8 h ‘asleep’ or in certain recovery stages of their sleep cycle in order to obtain the best levels of recovery and performance in their activity-dominated lives, they may become distressed by this available data. There is much variation in the accuracy with which such devices can accurately record sleep patterns. For example, many are negatively impacted by how much an individual moves or turns from one side of the body to the other during sleep (Lee et al., 2018). Therefore, instead of such recorded data being helpful, it can become a source of pressure and psychological distress for some individuals, perhaps then leading to an ironic, further disruption of, and negative impact on, the individual’s sleep patterns (Chen, 2019). App designers should attempt to refine their sleep monitoring features to address these limitations, or at a minimum, clearly state these limitations to their users, as many are not aware of them when they purchase the technology.

In addition to sleep monitoring, psychological state tracking, using various health-focused apps, has also become a feature of athlete screening in many sport settings in recent years, especially within elite domains. Some companies have incorporated health features into their wearable devices that provide stress level indicators to the user (such as sleep quality, exertion levels and sympathetic nervous system responses – including heart rate – for example). These indicators then provide the user with an overall ‘stress management score’ (Watters, 2020). These stress management indicators are then often followed by recommendations for sessions that users could engage in, using the same app, to help them positively adjust their mental states, and restore a degree of ‘balance’ to their physical and mental health levels, if deemed necessary. Fitbit, for example, has added a Stress Symphony musical feature to their health tracker offering to help wearers manage their stress levels (Fitbit Staff, 2020). In elite sport settings, support staff often ask athletes to rate their perceived fatigue levels pre-and-post training, their perceived effort after training, their mood state and their perceived pain levels, for example (in addition to such staff having access to GPS data that objectively measures some of the actual effort outputs of the athletes during the same training or competition sessions). Bush et al. (2019) reviewed some of the available literature on monitoring such psychological health features using various apps across various cohorts, considering specifically their use for self-management, supportive care, symptom tracking and skill training. They reported that while the benefits of these digital options include their ease of access and cost-effectiveness, empirical research regarding, for example, their more clinically targeted effectiveness is somewhat limited. However, that said, benefits for their use in the treatment of conditions such as depression (Watts et al., 2013), anxiety (Grassi et al., 2011), alcohol consumption (Fowler et al., 2016), and eating disorders (Juarascio et al., 2015) have been indicated. Smartphone technology has also been employed, for example, to assist in the detection of early warning signs regarding the onset of a manic or depressive episode in individuals diagnosed with bipolar disorder (Puiatti et al., 2011). Therefore, such technologies’ ability to assist in psychological screening on some level should not be dismissed outright. However, Bush et al. (2019) did also advise some notes of caution regarding their use, including the ‘overselling’ of their effectiveness by their designers (based upon insufficient clinical trials, for example), as well as data privacy and regulation issues.

Additional useful features of some smartphones and wearable devices are their abilities to record and present the amount of time individuals spend engaging in other online activities when using the devices (such as the amount of time spent emailing and using popular social media platforms for example, such as Instagram, Twitter, Facebook, and LinkedIn). These features can be beneficial for athletes to ‘track’ too, in order to help them remain aware of their schedules and see where they could be more productive in how they spend their time. During the pandemic, when typical social daily activities have been very limited, ways to fill daily schedules could be adapted and apps that could help the athletes and their support personnel to do this, such as by using calendars that synchronise across various online devices (i.e., smart phones, ipads, laptops, and desk-top computers) and among multiple-group-members were likely to have been useful.

In summary then regarding the above considered wearable health-related apps being used within sport settings, it is wise to remember that athletes by nature are typically competitive people, therefore, both they and their support personnel are advised to closely monitor the signs that they could be becoming pre-occupied with their app ‘data.’ Discussing openly the above cited potential dangers of such app use with them and giving honest advice regarding their beneficial use, as well as the limitations of such data monitoring features is advised (as outlined above). Once the users of such apps have the appropriate information regarding them they can then decide what data is helpful and unhelpful for them to have access to. They can also then aim to modify their behaviours accordingly where appropriate. For example, setting weekly and monthly targets that should not be exceed, as recommended above, can be an appropriate way to ensure at-risk athletes do not engage in excessive levels of activity to the detriment of their health and well-being. Encouraging them to engage, and indeed schedule in appropriate recovery and rest periods or days into their routines can be a way to effectively manage such situations (see Peake et al., 2018).

A final note of caution regarding any of the above technology devices that have the above features ‘build into them’ is, again, their reliability and validity. How accurately they gather the ‘correct’ and best data for athletes to have, with regard to their specific training and lifestyles remains somewhat questionable, from an empirical research standpoint (FITPRO, 2017; Bush et al., 2019; Nelson et al., 2020). The technology should be seen in many cases as only capable of providing a ‘guide’ and should not lead to athletes ignoring signals from their own bodies that may be telling them to rest or not to feel guilty about engaging, or not, in some behaviours, such as social media communications, in a fun way, for example, if their wellbeing could benefit from those activities (even if it does not seem like the most ‘productive’ way to spend their time according to others; The Drum, 2011).

## Online Sport Psychology Consulting During the Pandemic

Consulting online, prior to 2020, for many practitioners could have been the exception. However, in order to provide the necessary supports to athletes throughout the pandemic, ‘the exception’ became ‘the rule.’ This has not been unique for sport psychology practitioners, as cited earlier. Many other typically face-to-face service providers have had to make similar changes and adaptations to their practises (i.e., in settings such as general medicine, physiotherapy, coaching, and personal fitness training service delivery). For some practitioners, this was an easy transition because they were already offering some forms of online consulting prior to 2020. For others it has been a complete departure from their previous consultation practises. Such necessary changes may have generated great stress and unease for these individuals (Cotterill, 2020). For example, moving to an online service provision did not reduce the responsibility of such practitioners to maintain their professional and ethical standards. One worry for some service providers could have been the issue regarding online data or session information being accessed by unauthorised personnel. Such potential digital data security breaches are difficult to completely remove. Given the sensitive nature of some online psychology-based communications, such matters will likely remains a potential worry for practitioners, and their clients. This is often despite their best efforts to protect all parties involved by downloading various security and identification software features on the technology devices used to deliver such online services. For all of the above mentioned support service personnel, including sport psychologists, they have, despite these concerns, continued to provided people with much needed support services throughout the pandemic. Professional values have, hopefully, remained a priority in their online interactions with their patients and clients. For sport psychology practitioners specifically, based upon the regulations of their various societies [for example, the American Psychological Association (APA), the British Psychological Society (BPS), and the Psychological Society of Ireland (PSI)], they have endeavoured to keep in place their principles and values of: (i) beneficence (‘do good’ for their clients), (ii) cause no intentional harm to their clients, (iii) respect clients’ individual autonomy and independence, (iv) be fair, objective, caring, compassionate, responsible, and accountable (Etzel and Watson, 2017). Maintaining such standards when unable to engage in face-to-face, in-person, consultation does present some challenges, but not ones that are unmanageable.

First, however, one important question should be asked when service provisions for athletes move predominantly online: is a consultant ‘fit to practise’ using such online modes of communication? New technologies have traditionally been embraced by younger generations (Cotterill, 2012; Hurley, 2018). Being well-trained to provide any form of consultation service, both professionally and ethically, should be a key priority for all service providers. To their credit, organisations such as those cited above (BPS and PSI) have issued practitioners with guidelines and have held online training sessions to help their members ‘upskill’ in this regard (British Psychological Society [BPS], 2020; Psychological Society of Ireland [PSI], 2020). However, the primary responsibility lies with consultants, to allocate the time necessary to attend such training sessions and to attain the required skills in order to use the appropriate technologies professionally and correctly in their consulting practises. Assumptions regarding the digital skills and literacy of the athletes such professionals work with should also be considered in advance of using such online modes of consultation. To illustrate, when the pandemic brought about the closure of schools and colleges around the world, educators quickly had to adapt their teaching practises and ‘flip’ to an online mode of delivery (Mishra et al., 2020). There was a general assumption that the service providers (teachers and lecturers in this case) possessed all of the high-level technology skills in order to deliver the required material using this delivery mode, on such short-notice. Many people, such as the parents of children, and college students, not able to attend school or college physically during extended periods throughout 2020 due to various nations’ efforts to stop the spread of the coronavirus, soon discovered that this was not necessarily the case (Devitt et al., 2020). Expectations need to be managed regarding such online delivery of services in the future. That a consultant or athlete is an expert in using or managing all aspects of the available technology to consult online should not be assumed. Once honest communication is in place where both parties can feel safe in acknowledging their skill-set, could help both parties to engage in meaningful communications without unfavourable judgements happening about either party if something ‘goes wrong’ with the technology used during any online session. So what can go wrong, and what, specifically, what are the benefits, and threats, when providing such consulting services online?

## Benefits of Online Consulting

The obvious benefit of consulting online, or ‘remotely,’ is that it removes the location barriers that historically would have existed when athletes and their support staff could only interact if they were ‘physically’ in the same venue or location. Such remote communications throughout the pandemic, using technologies such as Zoom, Microsoft Teams, Skype, Facetime and Webex, have enable those involved in such service provision professions to remain safe from the threat of the coronavirus, as was the immediate priority from a public health perspective, while still being able to ‘do their job,’ most often from their own homes. The ability to record aspects of some online meetings and sessions using the available technology could also have been a benefit in some consulting environments, if deemed appropriate. Of course, this should only be used when all of the ethical issues around transparency, privacy and consent have been addressed prior to any recordings being taken.

## Threats of Online Consulting

Online consulting for sport psychologists has also brought with it all of the challenges that technology-use typically presents in any setting, including any of the mobile-Health (mHealth) settings, as cited earlier. For example, under General Data Protection Regulation (GDPR) laws, challenges regarding privacy and confidentiality issues in the management of digital personal data may have arisen for many service providers, as alluded to earlier. Along with those considerations, satisfactory internet connectivity is necessary for both parties to be able to engage in such online consulting service communications. Internet connectivity is notorious for its instability. It can be disrupted or not as well established via the infrastructure in some areas across different parts of the world, especially in more rural or remote locations. Digital illiteracy and digital poverty can also exist for practitioners and athletes in the same way as has been identified for many members of the general population throughout 2020 (Seah, 2020), as alluded to above also. Not all individuals have the required resources to also be able to afford the often expensive technology equipment needed to engage efficiently with the online world and to avail of all of its service offerings. During the pandemic, some sponsorship and ambassador roles with some of technology companies, who have thrived during this time, may have helped some athletes to overcome these specific barriers (Earley, 2020b). However, such opportunities for athletes has generally been the exception rather than the rule. It is not immediately apparent how many athletes have been disadvantaged, or ‘left behind,’ because they have been unable to avail of appropriate services throughout 2020 for the above cited reasons. This is an area requiring future research and resources to identify and address some of these issues.

Also, the importance of effective communication skills when consulting cannot be overstated. Consultants should have good listening skills, as well as the ability to interpret body language, including facial expressions and posture cues. Cotterill (2014) commented that anything other than face-to-face conversations are a compromise. Based on the way technology consulting typically operates, this is a very valid point. Full-body positioning is often not visible when using online consulting, in the way it would be in a face-to-face exchange (Smith, 2019). Even when the camera feature is enabled so that both parties can ‘see’ each other on an online screen, this is something not all individuals are comfortable with (as alluded to above when discussing the use of video technology in training for athletes). The ability to see the ‘full’ person is also not typical during such communications, due to the relatively small sizes of most computer screens, the positioning of the individuals ‘on-screen’ and the type of technology each person may have access to. There is also the frequent difficulty of a ‘time lag’ that occurs in many online video communications (The Irish Times, 2020). This can lead to both parties frequently ‘talking over’ one another. This tends to happen much less in face-to-face physical conversations because better conversational turn-taking ‘norms’ typically apply in such exchanges. Distractions, such as ‘outside’ noises, if individuals are not wearing earphones during any online discussion can occur also. The wearing of such earphones can be uncomfortable for many individuals if they are necessary for use over a lengthy period of time. This can be especially problematic if consulting is the fulltime position of the individual and if online sessions are held ‘back-to-back’ over several hours in a typical ‘working day’ (Cotterill, 2020). This can lead to specific health-related threats for online consulting and they are important consideration for all service providers when consulting online too. Regarding consultant availability, given that travel is typically not required for online sessions to take place if the consultant and client are both in their homes, there could be a mistaken view taken by the athletes, for example, that their consultants are available to ‘meet’ or chat with them online at any time. It is important to establish boundaries for availability, within certain daily work hours and to outline session length clearly in advance too. If not, the danger of sessions ‘running over’ and burnout or on-screen severe fatigue occurring is a definite possibility (Cotterill, 2020). Screen fatigue and ‘technostress’ (Brod, 1984; Brivio et al., 2018) is also a really danger for all individuals using online technology extensively throughout 2020, for work, education and training purposes. For example, headaches and eye strain can occur, as well as skeletal/orthopaedic issues (i.e., back pain resulting from long periods spent in a seated position, when forced to work from home). Some commercial opportunities for solutions to such issues have arisen as a positive outcome of these situations (Power, 2019). So, to conclude, as with any mode of service delivery, online sport psychology consulting has both benefits and challenges that the pandemic of 2020 has highlighted. However, sport psychology consultants must prioritise their own health and well-being first if they are to remain effective service providers for the individuals they seek to help, namely their athlete-clients (McCormack et al., 2015; Cotterill, 2020).

## Online Delivery of Physical and Psychological Training Programs, Including Remote Coaching

The coaching of athletes has also had to adapt during the pandemic. Many forms of online coaching have been happening throughout 2020 (Callary et al., 2020; Sorbie et al., 2020), with some positive outcomes reported regarding coach-athlete relationships (Li et al., 2020). Educational webinars and podcasts have also become popular, with coaches using the ‘lockdown’ periods to enhance their own professional development, as well as providing assistance to the athletes they work with. The importance of high quality instruction and communications during such sessions, as well as the value of the social support these methods of interaction provide has been apparent (Bailey, 2020; Evans et al., 2020). Some management teams and sport organisations have also used the pandemic time productively and creatively to collaborate with others in their sport networks in order to establish new, beneficial and closer relationships, as well as to avail of coach education expertise that prior to 2020, and often due to timings, availability and busy schedules may not have been possible (Bailey, 2020). Thus, the pandemic seems to have positively established a sense of ‘collaboration and community’ throughout those specific settings. The threat of the complete loss of positions and incomes within the world of sport for so many people has also been a possible ‘driver’ of these acts of collaboration (Lewis, 2020). Concerns over personal health, both physically and mentally, may have added to the burden many of these individuals have felt (Santi et al., 2020). They may have also been motivated to remain connected with more of the people from their own settings than before because they considered them perhaps better positioned to understand their worries, such as their concerns about their career trajectories and being less ‘essential’ during this time of great uncertainty (Taku and Arai, 2020). In many team sport settings, the importance of scheduling in time for online-team-coffee-meetups and devising team-building fun physical and mental challenges that could be globally bench-marked against others in their sport have been some other ways team members have endeavoured to maintain a form of connectivity with each other (McCarry, 2020; Whales et al., 2020). Social media use has also enabled such connections to remain in place throughout the pandemic.

Psychological interventions have also been delivered more frequently using various technologies over the past decade (Bush et al., 2019). Within sport settings, online interventions for self-talk (Latinjak et al., 2019), as well as imagery and goal setting (Lane et al., 2016) have been utilised with some success. Other commonly applied sport psychology strategies, for the enhancement of mental skills such as concentration and coping, have also been transferable to digital delivery modes, including diaphragmatic-breathing (Jerath et al., 2015); progressive muscle relaxation (Isa et al., 2013) and guided mindfulness meditations (Hoge et al., 2013).

In more clinically focused settings, technology-enhanced cognitive behavioural therapy has also been applied effectively to treat some mental health conditions across various populations (Twomey et al., 2013). Within sport settings specifically then, the online psychological intervention options, as referred to above, that have emerged in recent years could have been a welcomed option for athletes to utilise during the pandemic when in-person options of support from their sport science personnel were not available to many of them (Tayech et al., 2020). The effectiveness of such online service provision throughout the pandemic are likely to be the focus of much peer reviewed publications within the coming years and will be a welcome addition to the sport cyberpsychology literature.

## Athletes’ Use of Social Media During the Pandemic

Williams and Chinn (2010) stated that social media is comprised of “tools, platforms and applications that enable consumers to connect, communicate and collaborate with others” (p. 422). This type of technology is rapidly evolving. It provides athletes and sport organisations with unique and beneficial ways to interact with each other and their fan base (Hurley, 2018). Athletes are typically some of the most ‘followed’ individuals across many social media platforms. Indeed, during the 2020 pandemic, it has been widely reported that of the top three individuals most followed on social media platforms such as Instagram and Twitter, two are from the world of sport, namely, Cristiano Ronaldo (soccer) and Dwayne ‘The Rock’ Johnson (athlete, movie star and business man; with popstar, Ariana Grande, also in the 2020 ‘top-three’; Clement, 2020). The use of social media by athletes has been studied in some depth for approximately a decade and they have been found to utilise popular social media platforms and communities, such as Twitter, Instagram, Facebook, WhatsApp, and LinkedIn for a number of reasons, namely for: (i) personal communicating and socialising, (ii) fan engagement, (iii) professional networking, including brand management, ambassadorial roles and advertising opportunities, (vi) volunteering/charity work, and (v) political and societal influencing roles (Hambrick et al., 2010; Pegoraro, 2010; Hurley, 2018; Whales et al., 2020). From an elite perspective, knowing ‘best practise’ when using social media is important and sport organisation have also increased their training of their contracted athletes in this regard in recent years. They are now well aware, from past experiences in the world of sport, that any controversial communications online by their contracted athletes could be professionally and personally damaging for their club or organisation, as well as for the athlete and the sport concerned in general in the court of public opinion (Cotterill, 2019; ESPN, 2019; Express and Star, 2020). Public opinion has a high degree of power and athletes, when using the online, social media world should to be conscious of this, while also being encouraged to maintain a form of authenticity online, that is, of showing their ‘real’ selves in their online social media presentations. Authenticity and honesty in the online world is typically valued in a similar way as it is in the offline world (Francis and Hoefel, 2018). Research on the online representation individuals make of themselves in their online lives compared to their real, off-line lives typical shows that people, in general, opt to display themselves in a more favourable light online (Fullwood, 2015) and athletes are potentially no different in this regard (Hurley, 2018). Typically, their social media content displays activities from their sport and work/business lives. However, the pandemic has offered a unique opportunity for them to share more about areas of their lives outside of sport (Tayech et al., 2020). Sharpe et al. (2020) noted a specific change in ‘tone’ of the social media posts of many elite athletes during the pandemic, from one of commercial to one of altruistic intent. Sharpe et al. categorised the social media posts of the high-profile athletes they studies into three main types: (i) social and civic responsibility messaging, (ii) fundraising and physical activity challenges and (iii) financial generosity. While some could argue that these activities could lead to later commercial gains for the athletes involved, and indeed their sport organisations and affiliated teams, the definite change in focus way from predominantly sport participation to more every-day life activities has enabled athletes to interact with their fan base on a more personal and relatable note, which should be welcomed.

When isolated and forced to spend more time alone during 2020, for some athletes the use of their social media accounts has helped them to communicate and stay connected with the outside world – a positive employment of its use. Indeed, many athletes have the potential to be effective role-models during times of crisis (Leng and Phua, 2020). They have supported health-message campaigns during the pandemic via their social media posts, using their influence to engage with their fan-base and to help change behaviours, such as importance of the wearing of face coverings, hand washing and maintaining social distancing. Of course, social media also provided athletes with a platform to share their training schedules and training advice with their fellow athletes, support staff and the general public at-large (Tayech et al., 2020). This proactive and prosocial behaviour could have helped them to also maintain their own training motivation levels during a time when enthusiasm to keep training for competitions that were surrounded by uncertainty may have waned. However, other athletes may have opted to use the time during the pandemic to take some time away from their social media platforms in order to spend time with their families. They could focus on ‘nourishing’ themselves and their close relationships during this time (Schinke et al., 2020). They typically would not have been able to avail of such opportunities when working because, for many elite professional athletes especially, their roles require them to travel a lot and spend lengthy periods of time away from their homes and family environments. It would be interesting to complete some detailed, perhaps qualitative, research on the specific value of such increased ‘quality-time’ spent with family and friends that the pandemic has offered to many of these athletes and their support personnel. What positive changes the pandemic has brought about in such individuals’ lives, that they would like to see maintained into the future where possible when the pandemic has ended, could be a very worthwhile and interesting research undertaking (Whales et al., 2020).

## Some Conclusion and Final Thoughts: Future Possibilities Moving Forward

How athletes and their support providers continue to be positive, progressive and solution-focused as they ‘move forward,’ given the likely landscape of continued social distancing and restrictions on mass-sport-gathering into 2021 will be a significant challenge. However, 2020 has also enabled many of these individuals to, by necessity, become more skilled at conducting their interactions online and to use novel, safe and socially distant measures to engage in their training/occupation activities. These skills have become part of their ‘new normal’ ways of interacting with each other throughout 2020. As the pandemic continues into the near future, the required application of all of these measures should begin to feel less daunting, less ‘strange’ and more comfortable for those involved, until sport competitions and training environments can return to the way they were before the pandemic began. It will be interesting to see what the implications of the extensive online communications from 2020 will be for the legacy of future communication preferences, especially within such sport settings. Will there be an increased demand for more remote consultation practises in the years ahead? Could it become the preferred mode of consulting, the ‘new normal’? If anecdotal commentary is to be believed, online consulting is likely to continue alongside face-to-face sessions into the future, now that so many individuals working in these areas see the practical benefits such interactions offer, as another form of valuable service provision. However, the pandemic has also helped many athletes and their support team members realise the importance and tangible value of (i) ‘live’ sport events, with spectators present (Lewis, 2020), (ii) physical, social contact and (iii) in-person communications. Indeed, the human race cannot survive without people interacting in a real, physical way, nor can the economies of nations around the world survive in permanent restrictive, lockdown cycles, as has happened throughout 2020. ‘Living alongside covid-19,’ until an effective vaccine has been discovered, is the likely future for all populations. So while that is the case, to summarise in five main points, what specific avenues of novel research has 2020 potentially presented for individuals working specifically in the field of sport psychology?

First, as mentioned above, physical contact is one of the main things people have commonly reported missing the most throughout the pandemic (hand-shakes, hugging, and kissing, for example; McCluskey, 2020). Human beings are tactile and value physical interactions. Examining the impact of the necessary isolation periods on athletes and their support personnel, that may be somewhat different than those of the general public (Pietrabissa and Simpson, 2020), could be a fruitful research endeavour. Such explorations could help service providers to devise ways to ensure the mental and physical health of such individuals is prioritised in the months and years ahead. The restrictions currently in place in many countries mean that athletes can no longer to just ‘drop-in’ to see a service provider for such psychological support, or to stop for a chat in a corridor, for example. The physical social distancing requirement in many countries of two meters that helps individuals to be at a low-risk from contracting the coronavirus means that many individuals may not feel comfortable divulging personal difficulties they are experiencing when speaking to their service and support providers in the current ‘outdoor/apart’ settings because of the danger of others over-hearing their conversations. Such conversations would have been conducted in the past in a safe and private office space, for example. Continuing with online support services will help to overcome these issues in the short-term. However, more long-term supports and interventions are also likely to be needed to help many athletes and their support personnel exit the pandemic healthy and well, both mentally and physically.

Second, eencouraging athletes to engage in education-based training programs as the pandemic continues and evaluating such activities could also be a worthwhile endeavour, based upon some early research implications (Clemente-Suárez et al., 2020). The potential for such training and activities to not only improve athletes’ preparations for their lives after they retire from elite sport, but also to help them manage the uncertainty of their competition situation and their varying emotions about that while the pandemic continues, could be beneficial. Such education based training programs could help athletes to focus on other things inside of their control at this time and enhance their life skills, helping them to obtain other tangible gains in 2020 that, in more ‘normal’ times, may not have been possible due to their busy competition schedules (Clemente-Suárez et al., 2020; MacIntyre et al., 2020).

Third, evidence-based quantitative and qualitative research studies examining the detailed impact of the pandemic on the support providers working with athletes throughout this challenging time could be completed. More research is needed to objectively evaluate online consulting service provisions, including the technologies used to provide them. Such information could enable technologists and service providers to determine how such technology and services might be improved upon in the future. Research uncovering the specific stresses the pandemic has created for such service providers could also be beneficial, in order to help devise effective interventions to enable them to navigate successfully through the pandemic in a healthy and productive way. Support personnel should also be mindful that different athletes have different needs and the impact of the pandemic has highlighted that even more than before perhaps. Not all athletes have the technology skills necessary to use advanced equipment in their training when alone. Coaches and sport organisations need to be aware of this and educate them in order to change that situation moving into the future also. More government and technology-based company funding for technology provisions could also be helpful in this regard. How best to support athletes and sport organisations that do not have a high degree of technology expertise at present is unclear. Funding to implement an upgrading of their skills and the infrastructure needed to do this is also warranted. The pandemic’s more negative potential impact on certain athlete groups, such as Paralympic athletes, who may have had less access to the necessary equipment and training facilities in order to maintain their typical training regimes may also need to be highlighted and examined further (Clemente-Suárez et al., 2020).

Forth, for coaches and sport organisations specifically, maintaining some of their high-quality online coach training initiatives and knowledge-sharing could help them to maintain not only their own professional development but also solidify and strengthen those now established new collaborations and friendships that the pandemic has enabled them to forge across their sport communities. Such moves could benefit their skill-set as well as their wellbeing into the future. Research evaluating the approaches currently in place in this regard in order to ensure how best to achieve these aims into the future would also be welcomed. Fifth, advances in wearable technologies for performance tracking, as well as technology designed to enhance fan engagement and stadium experiences are areas that will likely continue to grow and evolve over the coming years (Low, 2020). Indeed, the inability of sport spectators around the world to physically attend sport events on mass, as could happen prior to the pandemic, makes this market and the demand for such technology even greater than before 2020. It can provide sport fans with some way to experience ‘real-life’ sport events when they are currently being mostly played ‘behind closed-doors’ and would likely be welcomed more than ever before by fans. This is a potential area for significant opportunity and investment by technology companies and researchers alike (Mayer, 2020; Miller, 2020). As discussed, wearable technologies have also been shown to be useful for mental skills training, as well as psychological state monitoring and the detection of early warning signs of psychological distress. Such technologies’ ability to provide psychological screening into the future, across various different cohorts, in even more effective ways is an exciting potential avenue of research. Designers and psychologists working together on such projects are often well positioned to help create the most effective psychologically based apps (Ryan et al., 2020). However, as Bush et al. (2019) also advised, for now, a cautious approach regarding their effectiveness is advised, with more clinical, controlled trials being needed to determine more definitively their best applications and practical uses.

To conclude, for so many people, when sport could not take place for the early months of 2020 especially, it highlighted the joy sport spectating and participation creates all over the world. It reinforced its important place in global society, as a priority, as efforts to ‘move forward’ despite the ongoing presence of the coronavirus. While the cyber world, and advanced technologies, will continue to evolve to the benefit, hopefully, of all athletes and their support personnel, no technology will replace the hard work, talent and determination of these individuals. The goal of technology and the cyber world to assist athletes in their preparations for their performances should always be athlete-welfare driven, with the very first principle being to ‘do no harm.’ Table 1 below presents a summary of some considerations for athletes and their support staff when using various technologies in the future, based upon their strengths and limitations/notes of caution, as presented in the discussion points raised throughout this narrative review.

**TABLE 1:** Some main considerations when using technology in sport settings in the future.

Video analysis technology	**Strengths:** (i) Source of objective biomechanical feedback for athletes and their support staff (i.e., for motor skill execution/technique; behaviour modelling opportunities) (ii) Potential source of intrinsic motivation for athletes for future sessions (iii) Beneficial uses for assisting in the decision making process of officials such as referees **Limitations/Notes of Caution:** (i) Feelings of pressure and perfectionism tendencies that may arise from the constant recording of training and competition performances
Virtual reality technology	**Strengths:** (i) Potential beneficial use during injury rehabilitation and for some mental skills training strategies **Limitations/Notes of Caution:** (i) Access difficulties to the most sophisticated technologies (ii) Limitations regarding the haptic experiences created by even the most advanced VR immersive technologies
GPS technology	**Strengths:** (i) Objective recordings of performance efforts, including the extent and directionality of various motor movements **Limitations/Notes of Caution:** (i) Feelings of pressure that may arise from the constant recording of training and competition outputs
Wearable technologies	**Strengths:** (i) Useful ‘measures’ of physical activity levels, psychological states, and sympathetic nervous system activity (i.e., heart rate and temperature) and sleep (ii) Potential source of intrinsic motivation for athletes for future sessions (iii) Potential recovery aid **Limitations/Notes of Caution:** (i) Questionable reliability and validity issues of the technology to provide truly accurate data, as is often required within elite sport, and medical settings specifically
Online sport psychology consulting/Online coaching delivery	**Strengths:** (i) Removal of location barriers to service provision when athletes and their support staff cannot be in physical contact (ii) Ease of access to, and affordability of, the required technology (i.e., Zoom, Facetime, and Skype) on devices most individuals have access to (such as laptops and mobile phones) **Limitations/Notes of Caution:** (i) Requirement of sufficient internet access to avail of such service provision (ii) Training requirements needed to use the technology effectively for such service provision (iii) Maintaining appropriate access boundaries/managing service provider potential burnout if always digitally ‘on-call’ (iv) Privacy and GDPR issues (v) Managing the expectations of all parties using such technology for online service provision (i.e., only certain behaviours may be visible as body cues, potential connectivity and ‘working from home’ disruptions)
Social media use	**Strengths:** (i) Source of helpful information related to training advice and behaviour modelling opportunities (ii) Source of social support – with other athletes, family, friends, and fans (iii) Specific, beneficial, uses for fan engagement, professional networking – including brand management and advertising opportunities, ambassadorial roles – including charity work, political, technological, and societal influencing roles (seen as beneficial throughout the pandemic for promoting health-related positive behaviours, including health-benefiting technology use) **Limitations/Notes of Caution:** (i) Inappropriate use (impulse control skills/social media training needed to use such media effectively and for positive forms of communication) (ii) Exposure to potential negative behaviours (online bullying, for example)

Over-burdening athletes with too much technology and data can result in increased stress and anxiety levels which their support team members should be cognisant of. Sometimes just relying on how athletes ‘feel’ after a training session, or competition can be more beneficial for their well-being than any data, especially in the midst of a pandemic. Ultimately technology, as a tool is neutral in essence, it is how people use it that determines if it is a positive or negative element in their lives. Time will tell how well the sport world uses technology for the good of all, as the COVID-19 pandemic continues, but hopefully comes to an end in the coming years when a safe and effect vaccine is produced and distributed.

## Author Contributions

The author confirms being the sole contributor of this work and has approved it for publication.

## Conflict of Interest

The author declares that the research was conducted in the absence of any commercial or financial relationships that could be construed as a potential conflict of interest.
